# Quantification of changes in balance control with tasks and injury using detrending methods for time series analysis

**DOI:** 10.3389/fbioe.2025.1589072

**Published:** 2025-06-24

**Authors:** Iva Radić, Suzana Blesić, Zdravko Aničić, Sladjan Milanović, Dragan M. Mirkov, Olivera M. Knežević

**Affiliations:** ^1^ Institute for Medical Research, University of Belgrade, Belgrade, Serbia; ^2^ Faculty of Sport and Physical Education, University of Belgrade, Belgrade, Serbia

**Keywords:** postural balance, lower extremity injuries, time series analysis, signal processing, scaling analyses

## Abstract

**Introduction:**

Human balance control is regulated by complex temporal processes that may be disrupted by injury or increased task difficulty.

**Methods:**

We examined long-range temporal characteristics of force platform recordings during quiet standing in 76 physically active participants with or without lower-limb injury, and in 13 non-injured participants standing with eyes closed or on one leg. Detrended fluctuation analysis (DFA) and wavelet transform spectral analysis (WTS) were used to quantify the temporal dynamics of postural control.

**Results:**

All recordings showed long-range autocorrelated behavior, with a visible crossover point separating random fluctuations at small time scales from structured dynamics at higher time scales (100 ms to 1 s). Changes in scaling behavior occurred only above the crossover point in response to altered stance or injury. Specifically, standing on one leg increased DFA and WTS slopes, likely due to enhanced amplitudes of characteristic peaks at approximately 250 ms and 650 ms. Two distinct postural responses to injury emerged: (1) compensation - characterized by increased amplitudes of all high-scale WTS modes and a crossover shift to smaller scales; and (2) underachievement - marked by decreased amplitudes and a shift of the crossover to larger time scales.

**Discussion:**

These findings support the potential of DFA, WTS, and similar time series techniques as sensitive tools for assessing subtle impairments in postural control.

## 1 Introduction

Participation in sports or recreational activities, besides the apparent health-related benefits, is also associated with a high risk of injuries, particularly in professional athletes (Emery and Pasanen, 2019). Most sport-related injuries are muscular or skeletal, affecting soft tissue, bones, ligaments, and nerves, thus reducing performance by causing muscle imbalances and asymmetries (Gimigliano et al., 2021). Although the type and intensity of muscle or motor capacity reduction depend on the type and severity of the injury, almost all of them affect the ability of athletes to maintain proper postural stability and balance. Since maintaining balance consists of movements around the ankle, knee, and hip joints and is regulated by the harmonized activity of corresponding muscles, injury, or reduction in the capacity of any of these muscles, can potentially affect the ability to control the balance efficiently (Panjan and Sarabon, 2010).

In general, balance can be considered locally as the ability of muscles to maintain equilibrium around single joints, or generally as the ability of body parts or of the overall body to achieve and maintain some steady state (Panjan and Sarabon, 2010; Winter, 1995). It can be described as static when the goal is to maintain the center of gravity (CoG) within the base of support with minimum movement, or dynamic when the task is to keep the stable position while performing given movements or tasks (Winter et al., 1990; Ricotti, 2011). Therefore, most of the methods used to assess balance have been focused on the trajectory of CoG, based on the idea that its variability is closely related to the ability to control the CoG over the base of support (Błaszczyk, 2016).

Thus, tests aimed to assess balance are usually based on tasks that challenge the control of CoG over the base of support while standing still on both legs and/or on one leg. Different approaches are used to quantify the amount, variation, and direction of the body sway (movement of CoG) or weight distribution between the legs (for a more detailed description of tests and derived variables please see (Panjan and Sarabon, 2010)). The rationale for assessment of balance as a part of performance examination among populations of athletes is based on the presumption that good balance represents one of the main prerequisites of an efficient athletic performance, but also on the fact that among other factors, good balance depends on the efficient synergistic action of the involved muscles (Behm and Anderson, 2006; Hrysomallis, 2011; Zemková, 2014).

In recent years, technological advances in rehabilitation—such as robotic-assisted systems, virtual reality environments, and wearable sensor technologies—have shown considerable promise in promoting motor recovery, particularly within gait training protocols (Ciobanu et al., 2018). While these approaches primarily target locomotor function, similar principles are increasingly being applied to postural balance rehabilitation, which remains the central focus of the present study.

Movements of the body or its CoG are nowadays most commonly measured on a force platform which measures the center of pressure (CoP) of the whole human body (Panjan and Sarabon, 2010). Records from a force platform are by nature complex outputs of fluctuating internal (body) drivers acting on different time scales. As such, they have been proven to be stochastic and long-range autocorrelated (Collins and De Luca, 1994), as are many other physiological records (Blesić et al., 1999; Milošević et al., 2002; Blesic et al., 2011; Stanley et al., 1999; Hausdorff et al., 2001; Wayne et al., 2013). In this paper, we examined long-range features of force platform data in quiet standing with or without injury, and in non-injured standing with eyes closed and on one leg only. To achieve this, we used the detrended fluctuation analysis (DFA) and the wavelet transform spectral analysis (WTS). DFA and WTS were applied to quantify the temporal dynamics of force platform data scaling exponents and characteristic times from their spectral decomposition, and to show how those parameters change with task or injury. It was already shown before that such time series parameters may provide a complementary and, in some instances, more sensitive and discriminating metric (compared to classical approaches) for characterizing human posture, and may serve as new indicators of change (Wayne et al., 2014).

The stochasticity of human quiet standing and in associated tasks or disorders has been assessed before (Collins and De Luca, 1994; Wayne et al., 2013; Wayne et al., 2014; Duarte and Zatsiorsky, 2001; Shimzu et al., 2002; Chagdes et al., 2009; Kirchner et al., 2012; Maze et al., 2016). Assessments of monofractal (including DFA) CoP characteristics of human postural sway have found that force platform recordings of quiet standing exhibit scaling behaviour, with crossovers in scaling and non-stationary dynamics (manifested, in the case of DFA scaling, in values of scaling exponents larger than 1) on time scales larger than 1 s (Collins and De Luca, 1994; Duarte and Zatsiorsky, 2001). To manage found data non-stationarity some of these researches additionally analysed time series of force platform increments (Kirchner et al., 2012), thus examining a more stationary data, or adopted the hypothesis that observed range of DFA exponents is within the range of error of the 1/f noise (Duarte and Zatsiorsky, 2001). Both DFA and spectral analysis of standing data found the crossover in CoP scaling to be in the time range between 10 ms and 1 s (Duarte and Zatsiorsky, 2001; Kirchner et al., 2012; Collins et al., 1995). It was shown that the position of the crossover changes with experimental conditions (such as standing with eyes closed or open), or with age (Kirchner et al., 2012). Additionally, studies have demonstrated that both monofractal and multifractal features of human quiet standing changed with training (Wayne et al., 2014), in the presence of balance disorders and pathologies (Maze et al., 2016), or with task and age (Chagdes et al., 2009). Finally, systematic assessments of surrogate data proved that the observed CoP behaviour was not a result of any feature of the methods used: DFA exponents of the shuffled surrogate sequences were around 0.5 for all subjects and conditions (Blesić et al., 1999; Kirchner et al., 2012). Namely, since all long-range correlations are destroyed by the shuffling procedure, the corresponding shuffled series will have scaling exponents of the random time series (Kantelhardt et al., 2001).

In this paper, we analysed recordings from two force platforms, to assess balance dynamics for each leg separately (Harrison et al., 2021). Due to the length of the recordings and constrains on data analysis posed by the finite size effects (see Data and Methods below), we analysed DFA and WTS features of time series in our dataset in time ranges of 2 ms to 1 s (or 1–500 Hz). This gave us insight into scaling behaviour in lower ranges of time scales compared to those reported in the literature to date (Duarte and Zatsiorsky, 2001; Kirchner et al., 2012). The inclusion of WTS analysis in our approach enabled identification of characteristic spectral (temporal) modes that constitute human posture in this time range.

Building on this methodological framework, the main motivation for this study is to enhance our understanding of how injuries and altered standing tasks affect the temporal dynamics of balance control in physically active individuals. Despite extensive existing research, the precise changes in balance control mechanisms following injury, as well as during challenging balance tasks, are not fully understood, particularly regarding the underlying temporal structure of postural sway.

Therefore, the specific aims of this study were: 1. To characterize and quantify the temporal scaling behavior of balance control signals (force platform data) using advanced time series analytical methods, namely, Detrended Fluctuation Analysis (DFA) and Wavelet Transform Spectral (WTS) analysis. 2. To identify how specific conditions (standing with eyes closed, standing on one leg only) and injuries (to the knee or ankle) alter the scaling dynamics, particularly focusing on long-range temporal correlations and characteristic spectral modes of sway.

We hypothesized that: 1. Changes in balance tasks (such as eyes-closed and single-leg stance) would lead to distinct alterations in scaling exponents and characteristic spectral peaks, indicative of modified neuromuscular control strategies. 2. Injuries to the lower extremities would result in identifiable patterns of compensation or underachievement in postural control, reflected through significant changes in the long-range temporal characteristics and spectral modes in the force platform data.

The findings from testing these hypotheses aim to demonstrate that DFA and WTS analyses could serve as more sensitive and informative tools for clinical assessments, rehabilitation monitoring, and injury prevention strategies by identifying subtle but significant shifts in balance control mechanisms that are typically undetected by conventional measures.

This paper is organized as follows: in the following section we provide essential information on experimental settings and participants to our experiments, together with a brief introduction to the two methods of time series analysis used. In the third section we describe our results. In the final section we provide discussion of our findings and propose directions for future research.

## 2 Materials and methods

### 2.1 Participants

Data from 93 physical active participants - 49 females (body weight 64 ± 7 kg; body height 174 ± 8 cm; age 22 ± 5) and 44 males (body weight 81 ± 8 kg; height 185 ± 7 cm; age 24 ± 2), were analysed in this study. All participants were physically active through their academic curriculum, which included approximately six to eight physical activity classes per week. Participants were free of any muscle-skeletal or neurological disease and had not taken any medications for at least 6 months prior to study participation. They were instructed to avoid any strenuous exercise for 2 days before the testing sessions. Prior to testing, participants were informed about the research purpose and procedures and provided written informed consent which was in accordance with the Declaration of Helsinki and approved by the University of Belgrade Institutional Review Board (02-273/21-1). Participants’ characteristics are summarized in Table 1. As part of the individual data collection, participants reported any history of knee, ankle, or other lower extremity injury.

**TABLE 1:** Characteristics of participants of standing recording.

Variable	10s Quiet standing		10s Eyes closed and one leg only standing	1.5s quiet standing before jump			
	Non-injured	Injured		Non-injured	Knee injury	Ankle injury	Other
N	8	5	8	40	22	10	4
Age (years)	20 ± 2	20 ± 2	20 ± 2	22 ± 5	22 ± 4	23 ± 5	22 ± 4
Gender (n)							
Male	2	1	2	21	9	6	2
Female	6	4	6	19	13	4	2
Injury side (n)							
Left	--	2	--	--	10	3	2
Right	--	3	--	--	12	7	2
Body height (cm)							
Male	187 ± 1	178	187 ± 1	182 ± 6	185 ± 8	189 ± 7	185 ± 1
Female	167 ± 11	169 ± 7	167 ± 11	171 ± 10	174 ± 10	172 ± 9	173 ± 2
Body weight (kg)							
Male	81 ± 1	80	81 ± 1	78 ± 9	85 ± 9	81 ± 8	80 ± 12
Female	71 ± 7	70 ± 4	71 ± 7	61 ± 7	67 ± 8	62 ± 8	61 ± 0.4
Physical activity (hrs/week)	8-10	8-10	8-10	8-10	8-10	8-10	8-10

### 2.2 Experimental procedures

Participants were instructed to stand still on two force platforms (AMTI BP600400, Advanced Mechanical Technology, Inc. Watertown, MA 02472–4800 United States) that sampled the horizontal (x and y) and vertical (z) components of the resultant ground reaction force at 1,000 Hz, separately for each leg. In the first experimental setting, 13 non-injured participants were required to perform three tasks that involved: 1) quiet standing with eyes open, 2) quiet standing with eyes closed, and 3) one-leg standing with arms relaxed (please see Table 1). After assuming the requested position, the task lasted for 10 s. A minute rest was given between the trials while rests between different tasks lasted 2 min.

In another set of experiments, 76 participants (see Table 1) were instructed to stand still on two force platforms for 2 s, as explained above, as an introduction to the countermovement jump exercise. The initial 1.5-s segments of the quiet standing from this experiment were used for time series analysis in this paper.

### 2.3 Data analysis

We used the detrended fluctuation analysis of the second order (DFA2) and wavelet transform spectral analysis (WTS) to investigate our data dynamics.

Detrended fluctuation analysis is a variant of a conventional fluctuation analysis, adapted for the analysis of non-stationary, non-linear data (Peng et al., 1994). In DFA, a fluctuation function $F\left(n\right)$ is calculated as a root mean square variation about the constantly changing local trend instead of the record’s mean, as is done in conventional fluctuation analysis (Peng et al., 1994). This is the first modification that DFA analysis utilizes on data series. It introduced the ‘detrending’ into the method, and ensures that, by calculating variation around the local trend, only intrinsic variations are examined by the method (Peng et al., 1994). In addition, $F\left(n\right)$ is calculated for a series of cumulative sum profiles $y_{i}=\sum_{k=1}^{i}x_{k}-\bar{x}$ (for i=1...N , N a number of data points in a data series, and $\bar{x}=1/N\;\sum_{k=1}^{N}x_{k}$ ) rather than the original record $x_{i}$ (Peng et al., 1994). This is the second modification that the method introduces to the original time series, and it arises from the original method design that stems from one-dimensional random walk theories (Peng et al., 1992). Namely, in the one-dimensional random walk theories it is the ‘walk’ rather than the current position of the data point that is of interest to the theory, which can it that way be derived from the methods developed in statistical physics (Peng et al., 1992). These two modifications are done as data pre-processing of the method. The detrended fluctuation function is then calculated as: $\left(n\right)=\sqrt{\frac{1}{\left(N-n+1\right)l}\sum_{i=1}^{N-n+1}\sum_{l=1}^{n}{y_{n,i}\left(l\right)}^{2}}$ . The local trend can be either a polynomial fit calculated on data segments of window size n , for $n=\left(1...N-1\right)$ (Kantelhardt et al., 2001), or a moving average calculated on window size n (Carbone, 2009). If the polynomial detrending is used, as we did in our analysis, the order of subtracted polynomial defines the order of the analysis (DFAm). It is considered that trends of order m−1 can be viewed as eliminated from the original record in DFAm (Kantelhardt et al., 2001; Kantelhardt et al., 2006).

The DFA method proved to be more stable than the conventional autocorrelation (ACF) or Fourier power spectra (PwS) analyses, with less noise and less pronounced finite-size effects (Bunde and Lennartz, 2012). It was shown in numerous repeated applications (Blesić, 2020), that due to the inherent power-law data dynamics in most of the real-world records - physiological and movement data included (Blesic et al., 2011) - $F\left(n\right)$ appears as $F\left(n\right)∼n^{\alpha }$ , a straight line on log–log plots of dependence of $F\;\left(n\right)$ of the time scale n . In such cases the slope α of this function, the DFA scaling exponent, is used to quantify the analysed series. It has been shown that 0.5 < α≤1 for stationary series (Peng et al., 1994), while the values α≥1 , which will be of partial interest to records used in this paper, imply the existence of intrinsic non-stationarities in data (Höll et al., 2016). In the latter case $F\left(n\right)$ usually exhibits crossovers, abrupt changes in log-log slope, while α≥1 may mean that the underlying process is of a composite nature (Höll and Kantz, 2015) that may result from an independent influence of different internal regimes on fast and slow time scales (Kantelhardt, 2009), or due to the interaction with correlated process or processes (Livina et al., 2003; Chen et al., 2002). Seemingly non-stationary behaviour can also result from the exceedingly high influence of periodic or quasi-periodic processes at some characteristic time or time scale (Blesić et al., 1999). If this is the case, these regular trends should be removed from the data prior to analysis (Kantelhardt, 2009). Finally, non-stationarities can be signs of an underlying multifractal dynamic (Kantelhardt, 2009).

**FIGURE 1:**
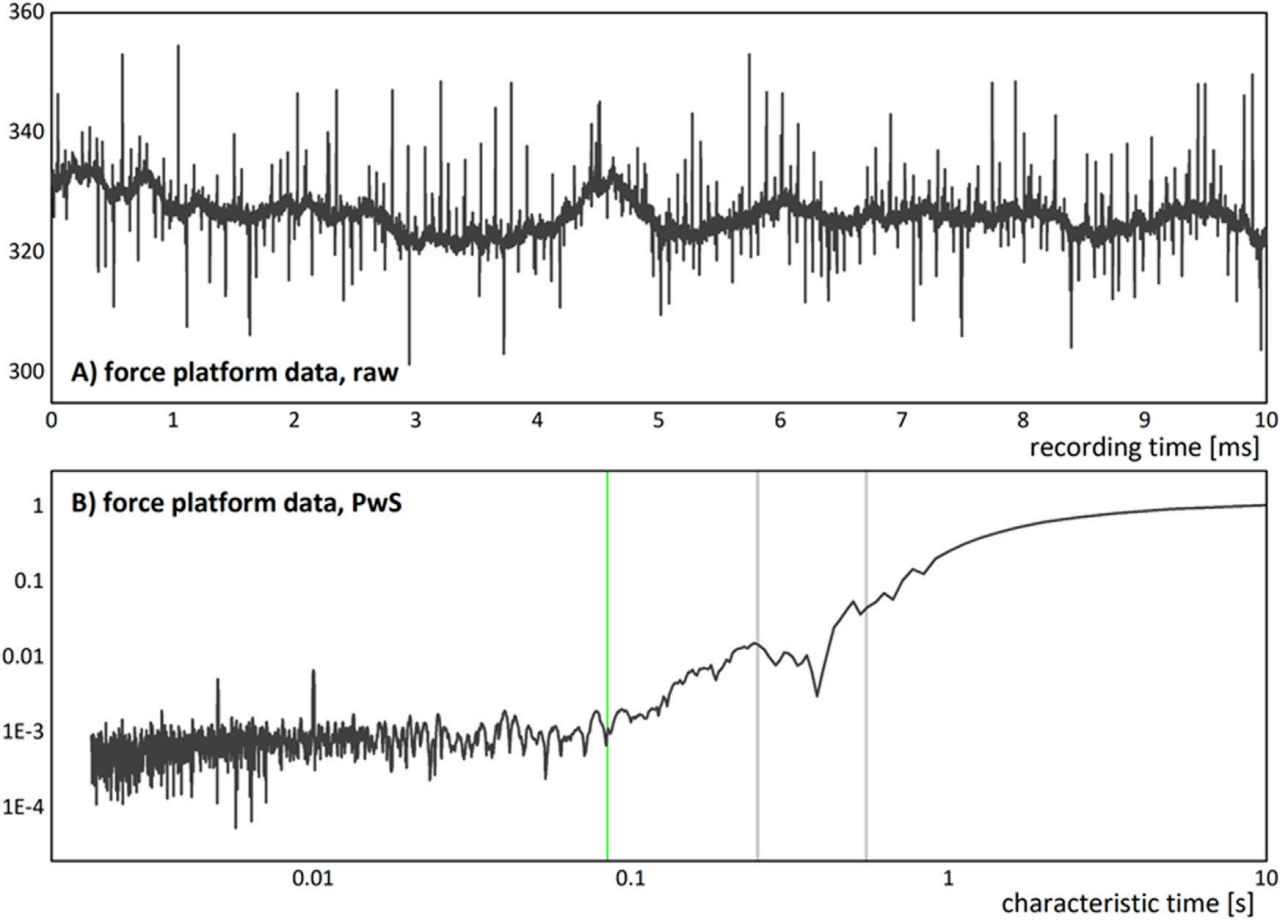
**(A)** An example of a force platform recording in quiet standing, of a z-axis (vertical direction) variations of the right leg. This raw data example shows noisy behaviour with visible multimodal variability (quasi harmonic behaviour of the low frequency underlying trend) which is present in all records in our dataset. **(B)** Log-log plot of Fourier power spectrum of the record in **(A)**, given as dependence of PwS on the characteristic time scale. The PwS presents with a possible crossover that is marked by a vertical green line, and two peaks after the crossover, at 250 and at around 550 ms, that are marked by the vertical light grey lines.

The advantages of using DFA and WTS over the more conventional statistical approaches (such as the calculation of Fourier power spectra) for the analysis of records from natural complex systems, including characterization of effects of tracking injuries are manyfold, and stem from the methods design (please see (Blesić et al., 2019) and references therein). The DFA, by way of detrending as preprocessing, produces a time series that fluctuates much less than the original, while preserving its statistical properties (Stanley, 2000). In this way it partially resolves the problem of direct calculations of the Fourier power spectra that are hindered by the level of noise present in a typical natural record (please see Figure 1B below). In this way DFA method provides a function that is allowing for clearer and less noisy presentation and interpretation of the results on log–log graphs.

Furthermore, pure long-range autocorrelated behavior rarely occurs in natural records. The corresponding DFA2 functions, depicted on the log–log graphs, tend to display transient crossovers in scaling that stem from occurrences of different intrinsic drivers of the analyzed behaviour (Blesić et al., 2019). When the effects of such irregularities are visible on DFA2 curves but are not comparatively strong to change the global behavior of DFA2 functions. We then use WT analysis to investigate them.

The wavelet transformation method was introduced to achieve signal localization and decomposition in both time and frequency (Wilczok, 2000). This method has been proven to possess the optimal joint time–frequency localization (Torrence and Compo, 1998) and can thus be used to effectively detect locations and spatial distribution of singularities in time series (Mallat and Hwang, 1992). Wavelet transformation is a two-dimensional time or space and scale decomposition of any signal or discrete series with functions constructed by expanding by time scale and translating along real time (or space) of a specifically chosen original wavelet function (Torrence and Compo, 1998). Decomposition along the real time in addition to time scale (analogues to frequency in Fourier analysis) allows for visualisation and inspection of local temporal components of the analysed signal (Torrence and Compo, 1998; Addison, 2018). This enables calculation of the local wavelet power spectra (lWTS), which are the localized contributions of the analysed time series energy at a specific time scale and at specific point in real time. Global wavelet power spectra $E_{W}\left(n\right)$ (WTS) are calculated when local wavelet power spectra are integrated over the real time (Torrence and Compo, 1998; Addison, 2018). WTS are mathematically comparable to Fourier power spectra (Perrier et al., 1995). For data with inherent power-law dynamics both WTS and PwS are of the power-law type, with the same power-law exponent β that can be related to the DFA2 exponent α through the scaling relation $\alpha =\left(\beta +1\right)/2$ (Peng et al., 1993). This makes two methods – DFA2 and WTS – comparable (Blesić, 2020).

To obtain statistically significant results and avoid effects of records' finite sizes on DFA2 statistics we calculated them between the time scales of n = 5 and n = N/5. Similarly, to obtain relevant statistical results for the WTS analysis, we calculated WTS functions between the time scales of n = 1 and n = N/5. In our dataset, with time series length of N = 10,000 data points for 10 s measurements or N = 1,500 for 1.5 s recordings, this limits time range of our investigations to $t_{\max}=2s$ ( $\left.t_{\max}=0.3s\right)$ . We used this scale range for visualization of our results. In drawing conclusions, however, we limited ourselves to a more rigorous, maximum statistically meaningful scale of $n_{\max}=N/10$ (Kantelhardt et al., 2006). The error to calculation of DFA2 exponents, which depends on N , is estimated from (Bashan et al., 2008), and in this paper equals to 0.05. Finally, to assess the significance of our WTS results we used tests of significance for detection of cycles in WTS (Torrence and Compo, 1998); we used this technique against the analysed signals as noise backgrounds.

## 3 Results

In Figure 1 we present one force platform record in quiet standing used in this study, in the form of raw data (Figure 1A) with their PwS spectrum (Figure 1B) given as a log-log plot of dependence on time scale instead of frequency (so that it could be compared with WTS spectra). The raw data show noisy behaviour with visible multimodal variability of the low frequency underlying trend that is present in all records in our dataset. The PwS spectrum of this record shows possible crossover in power-law (scaling) behaviour at time scales around 100 ms, with the probable existence of at least two peaks at the higher time scales, after the crossover.

**FIGURE 2:**
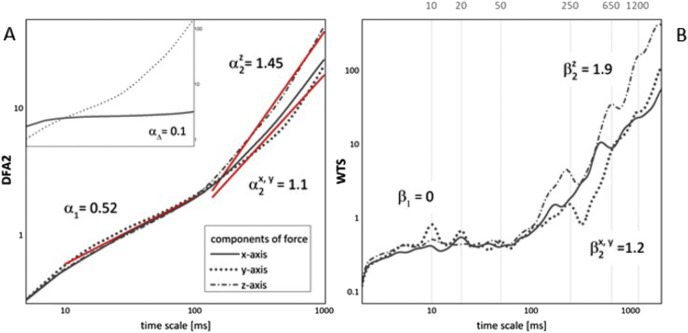
**(A)** DFA2 and **(B)** WTS functions of a typical non-injured record in standing, for one (right) leg and all three axes. In **(A)** slopes of DFA2 functions are given as red lines, with values of corresponding DFA2 exponents. Those are the same for all three axes below the crossover and are different for horizontal and vertical plain above the crossover. In the inset to **(A)** result of the DFA2 analysis for a series of increments (solid line) for z-axis are given in comparison to DFA2 result of the original series (dotted line), together with DFA2 exponent $\alpha_{∆}$ . In **(B)** values of WTS exponents are given for each function (axis), with vertical lines that serve as visual guides to examine characteristic peaks (modes) of muscle or movement activation.

In Figure 2 we present a typical result from our DFA2 and WTS analysis of standing still on a force platform data, for an individual who did not report recent injuries (from this point on, the ‘non-injured’ record or case). In all the force platform records that we analysed we found that DFA2 curves, as those depicted in Figure 2A, are approximately straight lines on log–log graphs. The scaling that we observed always exhibited crossover at timescales in the range of 50–100 ms, with scaling exponents below the crossover α_1_ equal to 0.5 (or $\beta_{1}\approx 0$ , for corresponding WTS functions), in all the analysed cases. This indicates completely random behaviour (distribution of force) in the small time scales area. In the larger time scales region, above the crossover, our non-injured records showed increase in slopes of DFA2 curves to values of $\alpha_{2}$ between 1.1 and 1.7 (that is, $1.2\leq \beta_{2}\leq 2.1$ ). Since values of $\alpha_{2}$ were larger than 1 in all our data, we performed DFA2 analysis on the series of increments ${∆x}_{i}=x_{i+1}-x_{i}\;\left(i=1...N-1\right)$ of the original series; if the original record has scaling exponent α>1 , the exponent of the series of increments ${∆x}_{i}$ should be $\alpha_{∆}=1-\alpha$ (Livina et al., 2003). We present this result in the inset of Figure 2A. It is apparent from this graph that series of increments of the force platform data do not show crossover in behaviour and that values of $\alpha_{∆}$ that we obtained are reduced by more than 1. This suggests that values of $\alpha_{2}>1$ in the region above the crossover point may be caused by a highly autocorrelated, non-trivial nonstationary regime.

The results of the global WTS analysis of a typical non-injured record, given in Figure 2B, show that WTS functions displayed existence of several characteristic peaks, or characteristic times of recorded behaviour, at 10, 20 and 50 ms in time ranges below the crossover point, and at around 250, 650 and 1200 ms, in higher time scales area. The amplitudes of WTS peaks were always more pronounced on z-axis than on the x- and y-axis. In addition, the scaling (DFA2 and WTS slopes) on x- and y-axis was always the same (within the range of error), which prompted us to focus on y- and z-axis only in our further analysis, assuming no loss of insight if we were to represent movements in horizontal plain by y-axis only.

**FIGURE 3:**
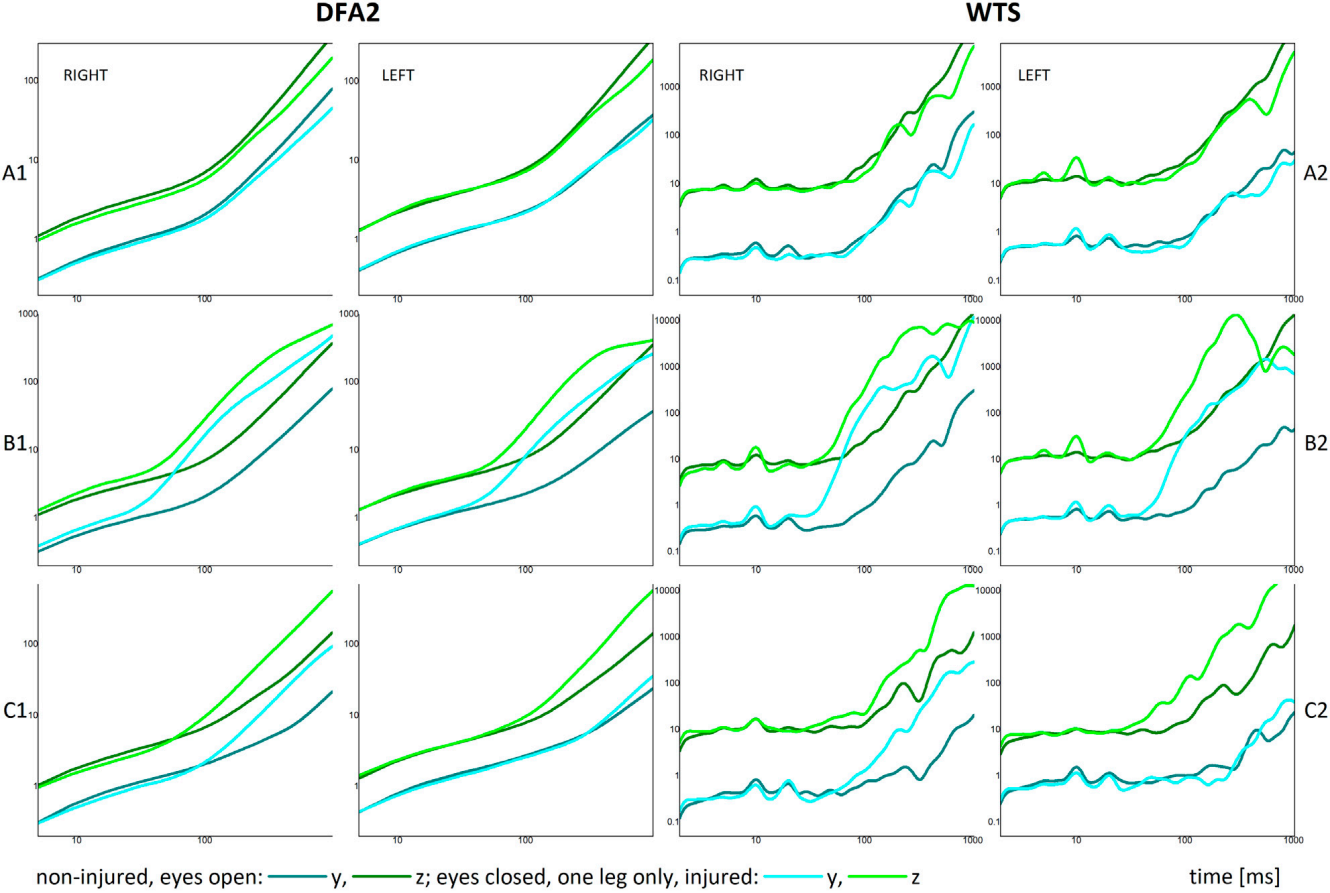
DFA2 and global WTS functions for three test conditions: **(A1,A2)** represent a non-injured person standing with eyes closed (compared to the eyes-open baseline); **(B1,B2)** show results for a non-injured person standing on one leg only (right or left leg); and **(C1,C2)** show results for a person with an injury to the left knee. Panels **(A1,B1,C1)** display DFA2 functions, while **(A2,B2,C2)** show the corresponding WTS functions.

To test how this behaviour changes with the loss of balance due to task or injury, we analysed several recordings of individuals who were not reporting injury and were standing on the force platform with their eyes closed and on one leg only, and of persons who reported injuries before or at the time of recording (data called ‘eyes closed’, ‘only right/left leg’, and ‘injured’ in what follows). Results of these analyses are shown in Figure 3, where the DFA2 and WTS functions for these different cases are given in comparison with the non-injured (and eyes open) record. Typical DFA2 results, presented in Figure 3A1,B1,C1, show clear change in the behaviour in non-injured standing on one leg and in cases of injury to one of the legs, in the higher time scales region, above the crossover point. There it is visible that the slopes of the DFA2 functions increase for both axes on the used-for-standing leg, when standing on one leg only (see Figure 3B1), or in the case of both axes for the uninjured (opposite) leg, and sometimes vertical (z-) axis for the leg with the reported injury (see Figure 3C1). DFA2 curves for the typical case of standing with eyes closed (Figure 3A1) do not show significant (within the range of error) change of slopes in comparison with the ‘eyes open’ case. Nevertheless, corresponding global WTS functions, shown in Figure 3A2,B2,C2, do show that even when DFA2 dynamics is unchanged, in the ‘eyes closed’ cases, the characteristic WTS peaks become more prominent in both small- and higher-time scales, while in cases of standing on one leg only or in cases of injury some of the peaks in the region above the crossover become increasingly prominent, and cause the observed significant change of the DFA2 and WTS slopes. It is also visible from Figure 3B2,C2 that the change of slopes (and thus the scaling) is caused by changes of amplitude of different characteristic temporal modes: for standing on one leg only (see Figure 3B2) significant change stems from increased amplitudes of peaks at around 250 and 650 ms, while in the case of injury given in Figure 3C2 the change is caused by activation of all the higher modes, including those larger than 1200 ms. Finally, it is visible from both DFA2 and WTS results how positions of crossovers shift to smaller time scales in all cases.

Of all the different settings presented in Figure 3, we further investigated how different kinds of injuries affect DFA2 and WTS results for force platform data. To this end, we analysed 29 records of upright standing in preparation for countermovement jump, in individuals who reported recent or current (related to the time of recording) injury to one leg. We compared their DFA2 and WTS results with the average DFA2 and WTS behaviour (that is, values of average slopes $\alpha_{1}$ and $\alpha_{2}$ , or $\beta_{1}$ and $\beta_{2}$ ) for the non-injured cases in the same dataset. As in previous cases, we did not find differences between injured and average non-injured dynamics in slopes of DFA2 or WTS functions in areas below the crossover point. We found differences in the higher times scales area; their values, defined as $∆\alpha_{2}=\alpha_{2,injury}-\alpha_{2av,non-injury},$ given as averages within groups of similar types of injuries, are reported in Table 2.

**TABLE 2:** Values of the difference Δα_2_ of the DFA2 slopes above the crossover point averaged over groups of data differentiated by the type of injury, given with the values of $∆\beta_{2}$ of the corresponding differences in WTS slopes. Values of Δα_2_ ( $∆\beta_{2}$ ) that are outside of the range of error are given in bold. The compensation discussed in the text is marked in blue.

Injury Location	Injury Type	Injured Side	Response – group average Δα_2_ ( $∆\beta_{2}$ ) (Standing)			
			right y	right z	left y	left z
Knee injuries	General	R	**−0.17 (-0.34)**	0.01 (0.02)	**−0.09 (-0.18)**	**−0.05 (-0.10)**
		L	**0.05 (0.10)**	**0.19 (0.38)**	0.00 (0.00)	0.02 (0.04)
	ACL	R	−0.04 (−0.08)	**0.18 (0.36)**	**0.10 (0.20)**	**0.13 (0.26)**
		L	**−0.16 (-0.32)**	0.00 (0.00)	**−0.27 (-0.54)**	**−0.14 (-0.28)**
	Severe	R	**−0.33 (-0.66)**	**−0.07 (-0.14)**	**−0.31 (-0.62)**	**−0.10 (-0.20)**
		L	**0.21 (0.42)**	**0.31 (0.62)**	**0.11 (0.22)**	**0.20 (0.40)**
Ankle injuries	General	R	**−0.05 (-0.10)**	0.03 (0.06)	−0.04 (−0.08)	0.01 (0.02)
		L	--	--	--	--
	Severe	R	**0.11 (0.22)**	**0.12 (0.24)**	−0.04 (−0.08)	**0.11 (0.22)**
		L	−0.04 (−0.08)	**0.13 (0.26)**	**−0.24 (-0.48)**	−0.03 (-0.06)

In Table 2 injuries of the knee are differentiated as ‘general’, in cases where we did not have specification of the types of injury, ‘ACL’, and ‘severe’ (such as dislocation or tear). It is important to note here that due to the small sample size all the reported statistics in Table 2 were calculated for 2–5 separate records in any injury groups and is thus generally poor; we present it only as a report, not as an indicator of any causation or statistical significance, and thus we are presenting just values averaged over the groups, without any error measurement. The only error that is presented here is the error to the DFA2 calculation that is due to the size of the data series N , which is explained in the Methods section of this paper. This is however a methodological, and not a statistical error.

All knee injuries in our sample show the similar kind of behaviour as the one depicted in Figure 3C2: the injury of one leg can lead to what we labelled ‘compensation’, or pronounced engagement of muscle groups or movement regimes that results in increased values of scaling exponents $\alpha_{2}$ , on both axes of the opposite (non-injured) leg and on the z-axis of the injured leg. Values of $∆\alpha_{2}$ for these injury groups, given in Table 2, suggest that this behaviour in our dataset may be present in ‘general’ knee injuries of the left leg and ACL injuries of the right leg. Contrary to the knee injuries, injuries of the ankle, distinguished in our sample as ‘general’ and ‘severe’ (such as break) (see Table 2), seem to lead to compensation on both axes of the same leg and on the vertical (z-) axis of the opposite leg, and only for injuries of the right leg. In addition to these responses, it is visible from Table 2 that some of the injuries of both knee and ankle can result in ‘underachievement’ above the crossover, which leads to lower values of α_2 than those of the average non-injured behaviour. In some of the cases this happens for both axes of both – injured and non-injured legs.

**FIGURE 4:**
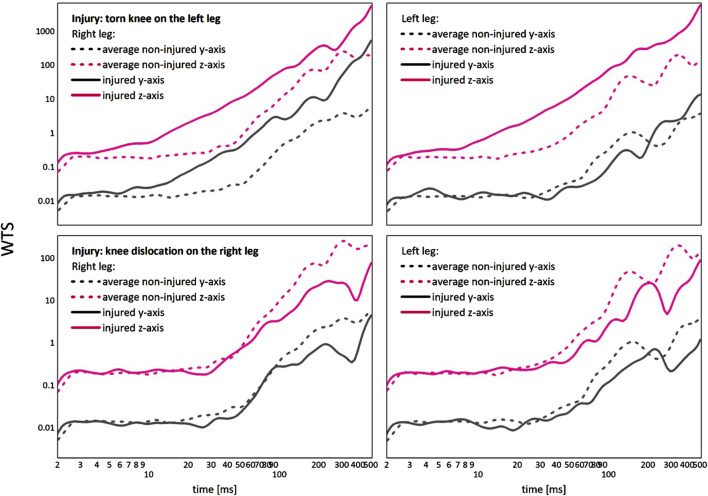
Comparison of “compensation” (upper row) versus the “underachievement” (lower row) behaviour, given as WTS functions in relation to the averaged non-injured WTS dynamics.

Examples of WTS functions of the ‘compensation’ on the opposite leg and ‘underachievement’ on both legs are given in Figure 4. We chose to use WTS functions for this illustration, for WTS functions are giving us access to not only data scaling (slope of the functions), but also characteristic peaks. The positions and the amplitudes of characteristic peaks of WTS functions may thus be used to understand the observed differences in scaling behavior with injury. To enable comparative assessment, we added the corresponding values of WTS exponents differences $∆\beta_{2}$ to Table 2 as well. These were calculated from the scaling equation that connects two scaling exponents α and β - $\alpha =\left(\beta +1\right)/2$ . Using WTS, it is visible from Figure 4 how ‘underachievement’ in our dataset is probably caused by the inability to engage muscles or muscle groups responsible for the characteristic peak at about 250 ms. It is also visible how this inability to engage may move the position of the crossover to higher time scales in cases of ‘underachievement’. On the other hand, the ‘compensation’, presented in the upper raw of Figure 4, is caused by increase of the amplitude of the characteristic peak at 250 ms, together with increase of the higher scale peaks that are outside of our range of analysis. This rise in peak amplitudes then shortens the range of scales before the crossover in behavior, compared to the averaged uninjured WTS behaviour.

## 4 Discussion

In this paper we analysed time series of force platform recordings of various tasks involving quiet standing, in individuals with or without injury to one leg, using DFA2 and WTS analyses. Our aim was to assess scaling dynamics in our dataset, to identify scaling and characteristic temporal spectral parameters, and to examine how those change in data recorded under different experimental conditions (standing with eyes closed or on one leg only), or with injury. We found scaling behaviour in all our data, with a visible crossover in scaling appearing at about 100 ms in non-injured quiet standing, a random behaviour on small time scales and a distinct non-linearity in long-term autocorrelated behaviour after the crossover area, on time scales in the range of 100 ms to 1 s. This result agrees with previous findings on CoP scaling (Collins and De Luca, 1994; Duarte and Zatsiorsky, 2001; Kirchner et al., 2012), in ranges of time scales where our research overlaps with the other groups. From WTS analysis we identified several characteristic peaks in scaling behaviour, at 10, 20 and 50 ms in the area below the crossover point, and at about 250, 650 and 1,200 ms in the area above the crossover. The characteristic modes that we found on higher time scales align with the frequency bands of the identified major modal scales around 3.03, 1.51 and 0.76 Hz in (Harrison et al., 2021) that were chosen as behaviourally relevant and not obscured by the measurement noise there. We distinguished found DFA2 exponents and WTS characteristic peaks as key parameters of our analysis.

Under the changes in experimental conditions or in injury to one leg, we found that only the scaling above crossover changes, while random behaviour below the crossover remains unchanged. In the case of standing with eyes closed we reported changes in the amplitudes of the characteristic peaks in both small scales and higher scales area that did not however led to change in the DFA2 slopes $\alpha_{2}$ . In cases of standing on one leg only and quiet standing with injury to one leg, we found an increase in slopes after the crossover point caused either by a visible change in amplitude of the characteristic peaks at about 250 and 650 ms (or around 4 and 1.5 Hz), in standing on one leg only, or by apparent continuous increase of amplitude of all the characteristic modes above the crossover point, including those outside the time scale range of our analysis, in injury. Increase in $\alpha_{2}$ was observed before as the increase of energy content at low frequencies (high time scales) while completing a cognitive task in quiet non-injured standing (Kirchner et al., 2012), or in the 0.049–100 Hz interval (that partially overlaps with our time scale range) for the eyes closed and tapping while standing tasks (Chagdes et al., 2009), with the effect more pronounced in older adults in eyes closed task and slightly less pronounced in the same population in tapping task. Some previous ‘eyes-closed’ results are dissimilar to our findings (Chagdes et al., 2009); further systematic assessment with the usage of same analysis techniques and in the same time scale areas may be done here by us, or by other groups. Finally, the increase of spectral energy content that was found before implies the shift in the position of the crossover point to smaller scales region (Kirchner et al., 2012), which is the result that we also report for standing on one leg only and in compensation for injury in our dataset.

Probably the most important finding of this paper is that DFA2 and WTS can distinguish injuries even in quiet standing, which corroborates claims that these and similar techniques may provide new, more sensitive discriminatory analysis framework for human balance assessments (Wayne et al., 2013; Wayne et al., 2014). We found two main types of response to injury: a) ‘compensation’, seen as the increase in the value of $\alpha_{2}$ , caused by the increase of all the characteristic modes above the crossover point that causes a shift of the position of crossover to smaller scales, and b) ‘underachievement’, which presents as a decrease of $\alpha_{2}$ , possibly caused by the decrease of amplitudes of the characteristic modes at around 250 and 650 ms that brings about a shift of the position of crossover point to higher time scales. Moreover, we found that injury to the knee, if compensated (in scaling behaviour), leads to compensation at the opposite (non-injured) leg and the vertical axis of the injured leg, while contrary the injury to the ankle results in compensation at the injured leg sometimes coupled with compensation at the vertical axis of the opposite leg, if compensation is present. This result needs further systematic assessment for different kinds of injury and balance disorders; it can nevertheless be already used to help understand the nature or sources of scaling and characteristic modes in the high scales area. Given the found difference in ways of compensation for the injuries of the ankle and of the knee, we can stipulate that our results may reflect the fact that injury to the ankle can still be compensated by elevated balance control at the hip of the same (injured) leg (Panjan and Sarabon, 2010; Winter et al., 1998), while the injury of the knee may result in compensation on/from the opposite leg as well. Finally, given the found increased engagement of characteristic WTS modes at about 250 and 650 ms in non-injured standing on one leg only and decreased engagement of those WTS modes in all “underachievement” leg injury responses, we may interpret this WTS change as an effect of engagement of specific muscle groups. If this is the case, we may speculate which of the muscles that contribute when stable one-leg stance is hypo-active (i.e., gluteus medius, tensor fasciae latae or lateral quadriceps) in this regard. For example, it has been shown that individuals undergoing or recovering from ACL reconstruction may have worse global dynamic postural stability compared with healthy control (Staples et al., 2020; Miko et al., 2021). This may represent the effect of the ACL injury or pre-existing deficits that contributed to the injury itself (Staples et al., 2020). Further work, by other groups or by us, on finding the links between WTS peaks and engagement or activation of some of the muscle groups or balance control strategies will represent an important way forward in understanding force development in standing, balance, and injury.

From our results it appears that the change of scaling in the horizontal plane is more discriminative to the injury type than the response in the vertical plane. It is important to note here that we chose to assess postural dynamics of all three axes separately for we showed in our previous work that the process of summation (such as determination of CoP) superposes different scaling and modal characteristics of the added signals, resulting in a DFA2 and WTS outputs dominated by the signals exhibiting higher autocorrelations (Blesić et al., 2019). It is already known that postural stability could be greatly impaired with respect to injury or illness (Błaszczyk, 2016; Staples et al., 2020; Miko et al., 2021); for example, anterior cruciate ligament reconstructed individuals have greater postural instability during the dual-cognitive condition that may indicate unique neural processing deficits remain following ACL reconstruction (Miko et al., 2021). Interestingly, similar patterns of postural stability were found before (Bodkin et al., 2018) in comparisons between individuals following ACL reconstruction and healthy individuals in a straight knee single leg balance task. That study concluded (Bodkin et al., 2018) that single-leg balance in a straight knee position may not be sensitive enough to detect impairments in ACL reconstruction patients at the time of return to sport progression. Although our injured participants had various lower-limb injuries, our study suggest that change of scaling could be sensitive enough to detect altered postural-control patterns.

Recent studies corroborate our findings: wavelet-based and nonlinear metrics capture subtle balance impairments during rehabilitation and sensory manipulations (Jafari et al., 2023; Czaplicki et al., 2017; Kodama et al., 2022; Piri et al., 2025).

Although formal clinical thresholds for DFA or WTS metrics do not yet exist, prior studies (e.g. (Duarte and Zatsiorsky, 2001; Kirchner et al., 2012; Maze et al., 2016)), have demonstrated that scaling exponents (α_2_) and wavelet power distributions differ systematically between healthy and pathological conditions, aging, and different task demands. This suggests that DFA and WTS have the potential to serve as sensitive, non-invasive tools in clinical settings for detecting subtle impairments in postural control. However, defining normative ranges and pathological cutoffs will require future studies.

Finally, our results do not settle the debate about the nature and origins of non-stationarity in force platform data. This remains to be further examined with access to longer time series (recordings) and/or utilization of various non-linearity tests. In that regard, a test of autocovariance difference (Höll et al., 2016) to assess whether values of $\alpha_{2}\;>\;1$ are due to the existence of intrinsic non-stationarities that were not removed from the data by the DFA algorithm could be utilized in the future for these types of records. Additionally, analyses of longer time series could unveil an imbalance between different noise inputs or activation modes that may be source of $\alpha_{2}\;>\;1$ as well. It can particularly enable examination of dominant WTS modes that arise from the quasi-periodic drivers of the entire body (such as breathing, heart rate, or metabolic processes) that we already found to influence dynamic properties of motor neuronal control (Blesić et al., 1999). This finding could then lead to removal of these dominant modes from the data and substantial lowering of value of the scaling exponent above the crossover point (Blesić et al., 2019).

### 4.1 Limitations

This study has several limitations. First, the duration of some force platform recordings was limited to 1.5 s, which may restrict the detection of lower-frequency sway dynamics and long-range scaling behavior. Studies suggest that shorter trial durations may not provide stable or reliable measurements of postural control, particularly for certain parameters sensitive to low-frequency sway. Longer trials, typically ranging from 60 to 150 s, are recommended to ensure the stability and reliability of posturographic assessments (Richmond et al., 2023; Amoud et al., 2007).

Second, injury data were obtained via self-report, introducing potential recall and classification bias. While this method is commonly used in sports and epidemiological research due to its practicality (Fuller et al., 2006), it may not accurately capture the precise timing, type, or severity of injury. Future studies by us or by other groups should aim to incorporate clinically verified diagnoses and longitudinal tracking to improve the accuracy of injury classification and its relationship with postural control adaptations (Fuller et al., 2006).

Finally, the most critical limitation lies in the small sample sizes within injury subgroups (n = 2–5), which limited the statistical power of group comparisons and precluded formal statistical significance testing or effect size estimation. As a result, we focused on descriptive findings and qualitative distinctions revealed through DFA and WTS methods, which remain sensitive to subtle alterations in balance control. While we fully acknowledge the importance of statistical measures in strengthening the validity of conclusions, reporting them under such constraints could be misleading. We have explicitly noted this limitation in the manuscript and emphasized the need for future studies with larger and clinically validated cohorts. Such research would enable more rigorous statistical evaluation and support broader generalization of our findings.

## Data Availability

The original contributions presented in the study are included in the article/supplementary material, further inquiries can be directed to the corresponding author.

## References

[B1] Addison P. S. (2018). Introduction to redundancy rules: the continuous wavelet transform comes of age. Philos. Trans. R. Soc. A Math. Phys. Eng. Sci. 376, 20170258. 10.1098/rsta.2017.0258 PMC6048575 29986912

[B2] Amoud H. Abadi M. Hewson D. J. Michel-Pellegrino V. Doussot M. Duchêne J. (2007). Fractal time series analysis of postural stability in elderly and control subjects. J. Neuroeng Rehabil. 4, 12. 10.1186/1743-0003-4-12 17470303 PMC1885443

[B3] Bashan A. Bartsch R. Kantelhardt J. W. Havlin S. (2008). Comparison of detrending methods for fluctuation analysis. Phys. A Stat. Mech. its Appl. 387, 5080–5090. 10.1016/j.physa.2008.04.023

[B4] Behm D. G. Anderson K. G. (2006). The role of instability with resistance training. J. Strength Cond. Res. 20, 716–722. 10.1519/R-18475.1 16937988

[B5] Błaszczyk J. W. (2016). The use of force-plate posturography in the assessment of postural instability. Gait Posture 44, 1–6. 10.1016/j.gaitpost.2015.10.014 27004624

[B6] Blesić S. (2020). Applications of statistical physics to study climate phenomena and contribute to overall adaptation efforts(a). Europhys. Lett. 132, 20004. 10.1209/0295-5075/132/20004

[B7] Blesic S. Maric J. Dragasevic N. Milanovic S. Kostic V. Ljubisavljevic M. (2011). Scaling analysis of bilateral hand tremor movements in essential tremor patients. J. Neural Transm. 118, 1227–1234. 10.1007/s00702-011-0581-1 21331462

[B8] Blesić S. Milošević S. Stratimirović D. J. Ljubisavljević M. (1999). Detrended fluctuation analysis of time series of a firing fusimotor neuron. Phys. A Stat. Mech. its Appl. 268, 275–282. 10.1016/S0378-4371(99)00110-7

[B9] Blesić S. Zanchettin D. Rubino A. (2019). Heterogeneity of scaling of the observed global temperature data. J. Clim. 32, 349–367. 10.1175/JCLI-D-17-0823.1

[B10] Bodkin S. G. Slater L. V. Norte G. E. Goetschius J. Hart J. M. (2018). ACL reconstructed individuals do not demonstrate deficits in postural control as measured by single-leg balance. Gait Posture 66, 296–299. 10.1016/j.gaitpost.2018.06.120 29958793

[B11] Bunde A. Lennartz S. (2012). Long-term correlations in Earth sciences. Acta Geophys. 60, 562–588. 10.2478/s11600-012-0034-8

[B12] Carbone A. (2009). “Detrending moving average algorithm: a brief review,” in IEEE Toronto International Conference Science and Technology for Humanity (Toronto, ON. Canada: IEEE). 10.1109/TIC-STH.2009.5444412

[B13] Chagdes J. R. Rietdyk S. Haddad J. M. Zelaznik H. N. Raman A. Rhea C. K. (2009). Multiple timescales in postural dynamics associated with vision and a secondary task are revealed by wavelet analysis. Exp. Brain Res. 197, 297–310. 10.1007/s00221-009-1915-1 19578840

[B14] Chen Z. Ivanov P. C. Hu K. Stanley H. E. (2002). Effect of nonstationarities on detrended fluctuation analysis. Phys. Rev. E 65, 041107. 10.1103/PhysRevE.65.041107 12005806

[B15] Ciobanu I. Stanculescu (Badea D. I. Iliescu A. Popescu A. M. Seiciu P. L. Mikolajczyk T. (2018). The usability pilot study of a mechatronic system for gait rehabilitation. Procedia Manuf. 22, 864–871. 10.1016/j.promfg.2018.03.122

[B16] Collins J. J. De Luca C. J. (1994). Random walking during quiet standing. Phys. Rev. Lett. 73, 764–767. 10.1103/PhysRevLett.73.764 10057531

[B17] Collins J. J. De Luca C. J. Burrows A. Lipsitz L. A. (1995). Age-related changes in open-loop and closed-loop postural control mechanisms. Exp. Brain Res. 104, 480–492. 10.1007/BF00231982 7589299

[B18] Czaplicki A. Kuniszyk-Jozkowiak W. Jaszczuk J. Jarocka M. Walawski J. (2017). Using the discrete wavelet transform in assessing the effectiveness of rehabilitation in patients after ACL reconstruction. Acta Bioeng. Biomech. 19, 139–146. 10.5277//ABB-00749-2016-02 29205225

[B19] Duarte M. Zatsiorsky V. M. (2001). Long-range correlations in human standing. Phys. Lett. Sect. A Gen. A. T. Solid State Phys. 283, 124–128. 10.1016/S0375-9601(01)00188-8

[B20] Emery C. A. Pasanen K. (2019). Current trends in sport injury prevention. Best. Pract. Res. Clin. Rheumatol. 33, 3–15. 10.1016/j.berh.2019.02.009 31431273

[B21] Fuller C. W. Ekstrand J. Junge A. Andersen T. E. Bahr R. Dvorak J. (2006). Consensus statement on injury definitions and data collection procedures in studies of football (soccer) injuries. Br. J. Sports Med. 40, 193–201. 10.1136/bjsm.2005.025270 16505073 PMC2491990

[B22] Gimigliano F. Resmini G. Moretti A. Aulicino M. Gargiulo F. Gimigliano A. (2021). Epidemiology of musculoskeletal injuries in adult athletes: a scoping review. A Scoping Rev. Med. 57, 1118. 10.3390/medicina57101118 PMC8539527 34684155

[B23] Harrison S. J. Kinsella-Shaw J. M. Dotov D. (2021). Effects of footedness and stance asymmetry confirm an inter-leg metastable coordination dynamics of standing posture. J. Mot. Behav. 53, 135–156. 10.1080/00222895.2020.1740151 32208833

[B24] Hausdorff J. M. Ashkenazy Y. Peng C. K. Ivanov P. C. Stanley H. E. Goldberger A. L. (2001). When human walking becomes random walking: fractal analysis and modeling of gait rhythm fluctuations. Phys. A Stat. Mech. its Appl. 302, 138–147. 10.1016/s0378-4371(01)00460-5 12033228

[B25] Höll M. Kantz H. (2015). The relationship between the detrended fluctuation analysis and the autocorrelation function of a signal. Eur. Phys. J. B (88), 1–7. 10.1140/epjb/e2015-60721-1

[B26] Höll M. Kantz H. Zhou Y. (2016). Detrended fluctuation analysis and the difference between external drifts and intrinsic diffusionlike nonstationarity. Phys. Rev. E 94, 042201. 10.1103/PhysRevE.94.042201 27841528

[B27] Hrysomallis C. (2011). Balance ability and athletic performance. Sport Med. 41, 221–232. 10.2165/11538560-000000000-00000 21395364

[B28] Jafari H. Gustafsson T. Nyberg L. Röijezon U. (2023). Predicting balance impairments in older adults: a wavelet-based center of pressure classification approach. Biomed. Eng. Online 22, 83. 10.1186/s12938-023-01146-3 37608334 PMC10463618

[B29] Kantelhardt J. W. (2009). “Fractal and multifractal time series,” in Encyclopedia of complexity and systems science (New York, NY: Springer New York), 3754–3779. 10.1007/978-0-387-30440-3_221

[B30] Kantelhardt J. W. Koscielny-Bunde E. Rego H. H. A. Havlin S. Bunde A. (2001). Detecting long-range correlations with detrended fluctuation analysis. Phys. A Stat. Mech. its Appl. 295, 441–454. 10.1016/S0378-4371(01)00144-3

[B31] Kantelhardt J. W. Koscielny‐Bunde E. Rybski D. Braun P. Bunde A. Havlin S. (2006). Long‐term persistence and multifractality of precipitation and river runoff records. J Geophys Res Atmos 111, 111. 10.1029/2005JD005881

[B32] Kirchner M. Schubert P. Schmidtbleicher D. Haas C. T. (2012). Evaluation of the temporal structure of postural sway fluctuations based on a comprehensive set of analysis tools. Phys. A Stat. Mech. its Appl. 391, 4692–4703. 10.1016/j.physa.2012.05.034

[B33] Kodama K. Yasuda K. Akatsuka T. Kuznetsov N. A. Iwata H. (2022). The influence of a vibrotactile biofeedback system on postural dynamics during single-leg standing in healthy older adults. Neurosci. Lett. 786, 136807. 10.1016/j.neulet.2022.136807 35850321

[B34] Livina V. Ashkenazy Y. Kizner Z. Strygin V. Bunde A. Havlin S. (2003). A stochastic model of river discharge fluctuations. Phys. A Stat. Mech. its Appl. 330, 283–290. 10.1016/j.physa.2003.08.012

[B35] Mallat S. Hwang W. L. (1992). Singularity detection and processing with wavelets. IEEE Trans. Inf. Theory. 38, 617–643. 10.1109/18.119727

[B36] Maze F. Blázquez-Tejada M. Rojas Ruiz F. (2016). Exploring body sway to disclose changes in postural control strategy associated with proprioceptive training. Eur. J. Hum. Mov. 37, 1–20.

[B37] Miko S. C. Simon J. E. Monfort S. M. Yom J. P. Ulloa S. Grooms D. R. (2021). Postural stability during visual-based cognitive and motor dual-tasks after ACLR. J. Sci. Med. Sport 24, 146–151. 10.1016/j.jsams.2020.07.008 32773174

[B38] Milošević S. Blesić S. Stratimirović D. J. (2002). Beneficial randomness of signals in a neuronal circuit. Phys. A Stat. Mech. its Appl. 314, 43–52. 10.1016/S0378-4371(02)01184-6

[B39] Panjan A. Sarabon N. (2010). Review of methods for the evaluation of human body balance. Sport Sci. Rev. 19, 19. 10.2478/v10237-011-0036-5

[B40] Peng C. K. Buldyrev S. V. Goldberger A. L. Havlin S. Sciortino F. Simons M. (1992). Long-range correlations in nucleotide sequences. Nature 356, 168–170. 10.1038/356168a0 1301010

[B41] Peng C. K. Buldyrev S. V. Goldberger A. L. Havlin S. Simons M. Stanley H. E. (1993). Finite-size effects on long-range correlations: implications for analyzing DNA sequences. Phys. Rev. E 47, 3730–3733. 10.1103/physreve.47.3730 9960430

[B42] Peng C. K. Buldyrev S. V. Havlin S. Simons M. Stanley H. E. Goldberger A. L. (1994). Mosaic organization of DNA nucleotides. Phys. Rev. E 49, 1685–1689. 10.1103/physreve.49.1685 9961383

[B43] Perrier V. Philipovitch T. Basdevant C. (1995). Wavelet spectra compared to fourier spectra. J. Math. Phys. 36, 1506–1519. 10.1063/1.531340

[B44] Piri M. Malmir K. Otadi K. Shadmehr A. (2025). Postural stability measures as diagnostic tools for chronic ankle instability: a comprehensive assessment. BMC Sports Sci. Med. Rehabil. 17, 16. 10.1186/s13102-025-01064-y 39885584 PMC11784114

[B45] Richmond S. B. Otto G. Dames K. D. (2023). Characterization of trial duration in traditional and emerging postural control measures. J. Biomech. 147, 111438. 10.1016/j.jbiomech.2023.111438 36641826

[B46] Ricotti L. (2011). Static and dynamic balance in young athletes. J. Hum. Sport Exerc 6 (6), 616–628. 10.4100/jhse.2011.64.05

[B47] Shimzu Y. U. Thurner S. Ehrenberger K. (2002). Multifractal spectra as a measure of complexity in human posture. Fractals 10, 103–116. 10.1142/S0218348X02001130

[B48] Stanley H. E. (2000). Exotic statistical physics: applications to biology, medicine, and economics. Phys. A 285, 1–17. 10.1016/S0378-4371(00)00341-1

[B49] Stanley H. E. Amaral L. A. N. Goldberger A. L. Havlin S. Ivanov P. Peng C. K. (1999). Statistical physics and physiology: monofractal and multifractal approaches. Phys. A Stat. Mech. its Appl. 270, 309–324. 10.1016/s0378-4371(99)00230-7 11543220

[B50] Staples J. R. Schafer K. A. Smith M. V. Motley J. Halstead M. Blackman A. (2020). Decreased postural control in patients undergoing anterior cruciate ligament reconstruction compared to healthy controls. J. Sport Rehabil. 29 (29), 920–925. 10.1123/jsr.2019-0154 31689685

[B51] Torrence C. Compo G. P. (1998). A practical guide to wavelet analysis. Bull. Am. Meteorol. Soc. 79, 61–78. 10.1175/1520-0477(1998)079<0061:apgtwa>2.0.co;2

[B52] Wayne P. M. Gow B. J. Costa M. D. Peng C. K. Lipsitz L. A. Hausdorff J. M. (2014). Complexity-based measures inform effects of Tai chi training on standing postural control: cross-Sectional and randomized trial studies. PLoS One 9, e114731. 10.1371/journal.pone.0114731 25494333 PMC4262457

[B53] Wayne P. M. Manor B. Novak V. Costa M. D. Hausdorff J. M. Goldberger A. L. (2013). A systems biology approach to studying Tai chi, physiological complexity and healthy aging: design and rationale of a pragmatic randomized controlled trial. Contemp. Clin. Trials 34, 21–34. 10.1016/j.cct.2012.09.006 23026349 PMC3638751

[B54] Wilczok E. (2000). New uncertainty principles for the continuous gabor transform and the continuous wavelet transform. Doc. Math. 5, 207–226. 10.4171/dm/79

[B55] Winter D. A. (1995). Human balance and posture control during standing and walking. Gait Posture 3 (3), 193–214. 10.1016/0966-6362(96)82849-9

[B56] Winter D. A. Patla A. E. Frank J. S. (1990). Assessment of balance control in humans. Med. Prog. Technol. 16, 31–51. 2138696

[B57] Winter D. A. Patla A. E. Prince F. Ishac M. Gielo-Perczak K. (1998). Stiffness control of balance in quiet standing. J. Neurophysiol. 80, 1211–1221. 10.1152/jn.1998.80.3.1211 9744933

[B58] Zemková E. (2014). Sport-specific balance. Sport Med. 44, 579–590. 10.1007/s40279-013-0130-1 24293269

